# Effect of *n*-3 (Omega-3) Polyunsaturated Fatty Acid Supplementation on Metabolic and Inflammatory Biomarkers and Body Weight in Patients with Type 2 Diabetes Mellitus: A Systematic Review and Meta-Analysis of RCTs

**DOI:** 10.3390/metabo11110742

**Published:** 2021-10-28

**Authors:** Leila Khalili, Roxana Valdes-Ramos, Laurence S. Harbige

**Affiliations:** 1 Department of Community Nutrition, Faculty of Nutrition and Food Sciences, Tabriz University of Medical Sciences, Tabriz 51368, Iran; 2 Lider del Cuerpo Academico de Nutricion y Salud, Facultad de Medicina, Universidad Autonoma del Estado de Mexico, Paseo Tollocan, esq. Jesus Carranza, Col. Moderna de la Cruz, Toluca 52180, Mexico; rvaldesr@uaemex.mx; 3 Lipidomics and Nutrition Research Centre, London Metropolitan University, 166-220 Holloway Road, London N7 8DB, UK

**Keywords:** meta-analysis, *n*-3, polyunsaturated fatty acids PUFA, T2DM, diabetes, randomized controlled trials RCTs, glycemic control, metabolic and inflammatory biomarkers, weight, BMI

## Abstract

Beneficial effects of *n*-3 fatty acids on metabolic biomarkers in patients with type 2 diabetes (T2DM) has been reported. The objectives of this current research were to investigate the effects of *n*-3 supplementation on metabolic factors, weight, and body mass index (BMI) in patients with type 2 diabetes mellitus (T2DM), using a meta-analysis of randomized, controlled trials (RCTs). Online databases PubMed, Embase, Web of Science, and Science Direct were searched until 2021 to identify eligible articles. Thirty trials were included. The results showed that *n*-3 consumption can significantly reduce glycemic factors including fasting blood sugar (FBS) (−0.36 (−0.71 to −0.01)), glycated hemoglobulin (HbA1c) (−0.74 (−1.13 to −0.35)), and homeostatic model assessment of insulin resistance (HOMA.IR) (−0.58 (−1.13 to −0.03)). Furthermore, significant improvement in lipid profile including triglycerides (TG) (−0.27 (−0.37 to −0.18)), total cholesterol (−0.60 (−0.88 to −0.32)), low density lipoprotein (LDL) (−0.54 (−0.85 to −0.23)), and high-density lipoprotein (HDL) (0.60 (0.23 to 0.96)) levels were found in the present meta-analysis. The reduction in the inflammatory marker’s tumor necrosis factor-alpha (TNF-α) (−0.13 (−0.75 to 0.48)) and c-reactive protein (CRP) (−0.72 (−1.70 to 0.27)), as well as weight (−0.09 (−0.24 to 0.07)) and BMI (−0.13 (−0.29 to 0.02)) were not statistically significant. Furthermore, the findings revealed that the optimal dose and duration of *n*-3 consumption for patients with T2DM is 1000–2000 mg/d for more than 8 weeks. The present meta-analysis and review reveals that *n*-3 supplementation can improve glycemic factors and lipid profile in patients with T2DM. Furthermore, *n*-3 supplementation may provide beneficial effects on inflammatory markers and body weight if used at the appropriate dose and duration.

## 1. Introduction

Type 2 diabetes (T2DM) is a metabolic disorder characterized by hyperglycemia in the context of insulin resistance and β-cell dysfunction. Its prevalence is increasing at an alarming rate worldwide [1,2]. Epidemiological and clinical trials have demonstrated that lifestyle, in particular daily diet, is of importance in the development and treatment of T2DM [3]. 

It has been reported that high fish and seafood consumption can significantly reduce the incidence of T2DM [4]. Bang et al. attributed such benefits of fish consumption to its main components, *n*-3 fatty acids (in particular eicosapentaenoic acid (C20: 5 *n*-3, EPA) and docosahexaenoic acid (C22: 6 *n*-3, DHA)) a family of homologue polyunsaturated fatty acids (PUFAs) [5].

Recent data supports the beneficial effects of *n*-3 PUFAs intake on metabolic profiles in patients with T2DM and obesity [6], and gestational diabetes (GDM) [7]. Several studies have reported that circulating levels of *n*-3 fatty acid were negatively associated with the risk of T2DM [8,9]. Moreover, some studies have demonstrated that *n*-3 fatty acids administration improve insulin sensitivity in overweight women with an inflammatory phenotype [10], whereas others observed that *n*-3 fatty acids in healthy people have no insulin-sensitizing effects [11]. Interestingly, favorable effects of *n*-3 fatty acids on inflammation and oxidative stress have been reported in patients with a high inflammatory status including pregnant women with GDM [12] and cancer-related cachexia [13]. These discrepancies may be explained by the different population studies, the origin of *n*-3 fatty acids, dosage, and length of treatment. Considering that the pathogenesis of T2DM may be linked to impaired metabolic profiles, inflammation and oxidative stress and as there is evidence that *n*-3 fatty acids may have anti-inflammatory effects and reduce oxidative stress [12], several trials have been conducted evaluating the beneficial effect of *n*-3 polyunsaturated fatty acids (PUFA) consumption on metabolic parameters in patients with T2DM. 

The purpose of the present study was to perform a systematic review and meta-analysis of randomized controlled trials (RCTs) on the effects of *n*-3 PUFAs, both the parent alpha-linolenic acid (ALA, C18: 3 *n*-3) and the longer chain *n*-3 PUFA EPA and DHA, on metabolic biomarkers including glycemic profile, lipid profiles, and inflammatory parameters and body weight and body mass index (BMI) in patients with T2DM. 

## 2. Results

**Figure 1:**
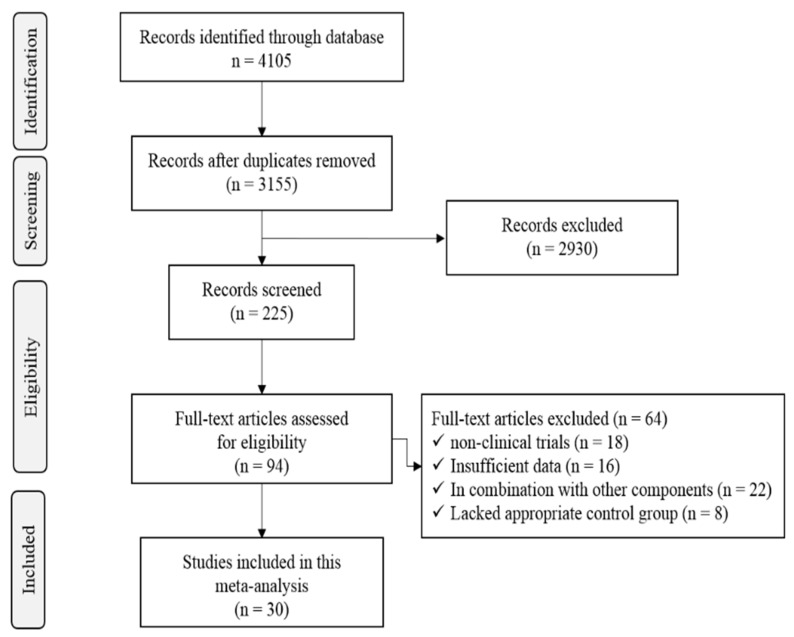
PRISMA flow chart of study selection process.

The literature search yielded 4105 citations. We retrieved 94 articles, of which 30 met eligibility criteria. A flow chart on article selection for the meta-analysis is shown in Figure 1.

### 2.1. Characteristics of Included Studies 

Table 1 contains specific information on intervention dosages and duration of treatment, and measurement parameters in studies. Thirty randomized clinical trials were included in the final meta-analysis. Twenty-four studies reported changes in glycemic factors, 20 studies reported changes in lipid profile, 13 studies reported changes in body weight, and 6 studies reported changes in inflammatory markers (Table 1). The subjects of all studies consumed *n*-3 as oral supplementation.

### 2.2. Meta-Analysis Results 

#### 2.2.1. Glycemic Factors 

##### FBS

**Figure 2:**
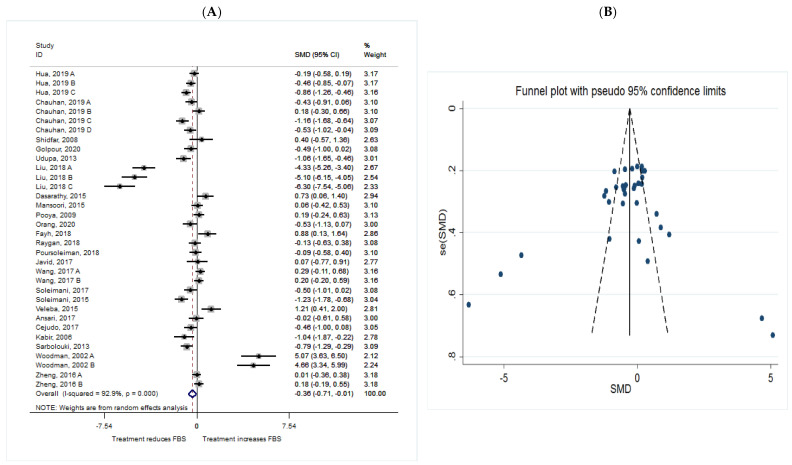
(**A**) The effect of omega-3 on FBS; (**B**) funnel plot examining the publication bias of FBS and *n*-3 supplementation.

The common SMD from 24 studies was −0.36 (95% CI: −0.71 to −0.01, *p* = 0.047) based on a random effect model, with significant heterogeneity between studies (I^2^ = 92.9%, *p* = 0.00) (Figure 2A). Further investigation detected 7 outliers with large effect sizes (Liu, 2018 A, SMD = −7.17; Liu, 2018 B, SMD = −8.57; Liu, 2018 C, SMD = −10.73; Fayh, 2018, SMD = 2.24; Veleba, 2015, SMD = 2.84; Woodman, 2002 A, SMD = 9.81; Woodman, 2002 B, SMD = 9.07), so we removed them from further analyses in this outcome. Removing these studies resulted in a reduction in effect size (SMD = −0.28, 95% CI: −0.46 to −0.10), but heterogeneity still remained significant although considerable reduction in heterogeneity was observed (I^2^ = 72.2%, *p* = 0.00). According to the shape of the funnel plot, we considered that there was no obvious publication bias among the included studies (Figure 2B).

##### HbA1c

**Figure 3:**
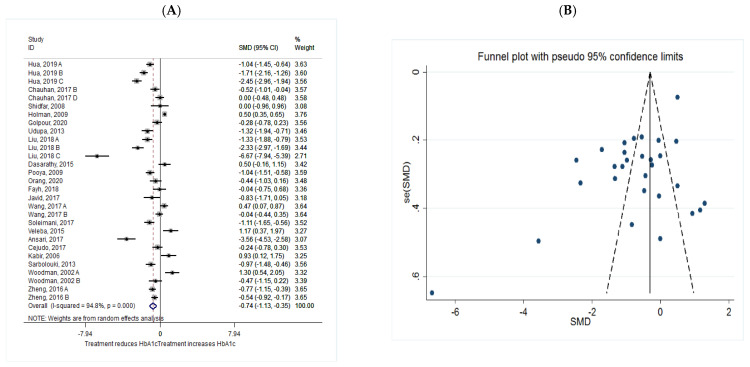
(**A**) The effect of *n*-3 on HbA1c; (**B**) funnel plot examining the publication bias of HbA1c and *n*-3 supplementation.

The common SMD from 21 studies was −0.74 (95% CI: −1.13 to −0.35, *p* = 0.00) based on a random effect model, with significant heterogeneity between studies (I^2^ = 94.8%, *p* = 0.00) (Figure 3A). Further investigation detected 9 outliers with large effect sizes (Hua, 2019 C, SMD = −2.45; Holman, 2009, SMD = 0.5; Liu, 2018 B, SMD = −2.33; Liu, 2018 C, SMD = −6.67; Dasarathy, 2015, SMD = 0.5; Soleimani, 2017, SMD = −1.11; Veleba, 2015, SMD = 1.17; Cejudo, 2017, SMD = 2.55; Sarbolouki, 2013, SMD = −0.97), thus these were removed from the analyses in this outcome. Removing these studies resulted in a reduction in effect size (SMD = −0.55, 95% CI: −0.90 to −0.20), but heterogeneity remained significant although considerable reduction in heterogeneity was observed (I^2^ = 88.5%, *p* = 0.00). According to the shape of funnel plot, we considered that there was no obvious publication bias among the included studies (Figure 3B).

##### HOMA.IR

**Figure 4:**
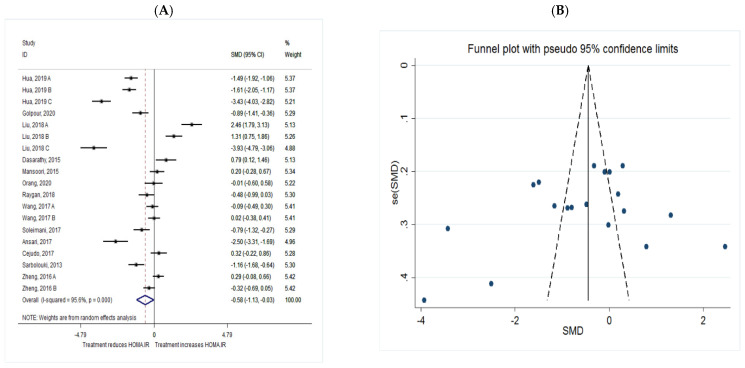
(**A**) The effect of *n*-3 on HOMA.IR; (**B**) funnel plot examining the publication bias of HOMA.IR and *n*-3 supplementation.

The common SMD from 13 studies was −0.58 (95% CI: −1.13 to −0.03, *p* = 0.038) based on a random effect model, with significant heterogeneity between studies (I^2^ = 95.6%, *p* = 0.00) (Figure 4A). Further investigation detected 5 outliers with large effect sizes (Hua, 2019 C, SMD = −3.28; Liu, 2018 A, SMD = 3.50; Liu, 2018 B, SMD = 2.17; Liu, 2018 C, SMD = −3.85; Ansari, 2017, SMD = −2.21), these were therefore removed from any further analyses. This resulted in a reduction in effect size (SMD = −0.38, 95% CI: −0.75 to −0.01), but heterogeneity remained significant although considerable reduction in heterogeneity was observed (I^2^ = 88.5%, *p* = 0.00). According to the shape of funnel plot, we considered that there was no obvious publication bias among the included studies (Figure 4B).

Sub-group analysis of different dosage of *n*-3 (<1000, 1000–2000, and >2000 mg/d) showed that *n*-3 consumption of 1000–2000 mg/d could significantly reduce FBS level and HOMA.IR; however, the reduction in supplementation for less than 1000mg/d and more than 2000 mg/d was not significant. The reduction in HbA1c level was significant at all the 3 dose sub-groups. Moreover, sub-group analysis of duration of *n*-3 consumption (≤8 week/>8 week) showed that *n*-3 consumption for more than 8 weeks could significantly reduce FBS level and HOMA.IR, but the reduction in HbA1c level was significant at both ≤8 and >8 weeks (Table 2).

#### 2.2.2. Lipid Profile

##### TG

**Figure 5:**
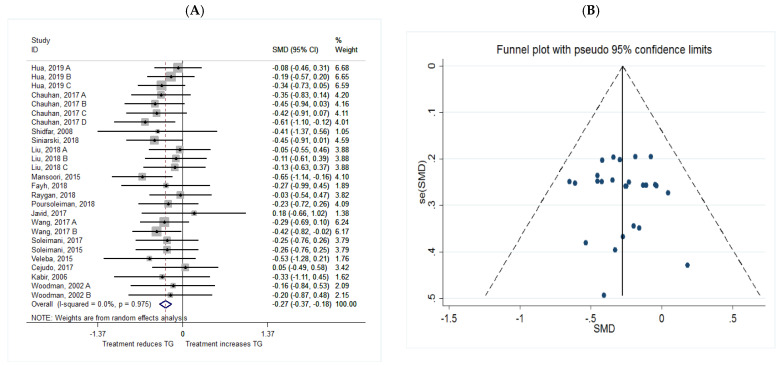
(**A**) The effect of *n*-3 on TG; (**B**) funnel plot examining the publication bias of TG and *n*-3 supplementation.

The common SMD from 17 studies was −0.27 (95% CI: −0.37 to −0.18, *p* = 0.00) based on a random effect model, with no heterogeneity between studies (I^2^ = 0.0%, *p* = 0.975) (Figure 5A). Furthermore, according to the shape of funnel plot, we considered that there was no obvious publication bias among the included studies (Figure 5B).

##### Cholesterol

**Figure 6:**
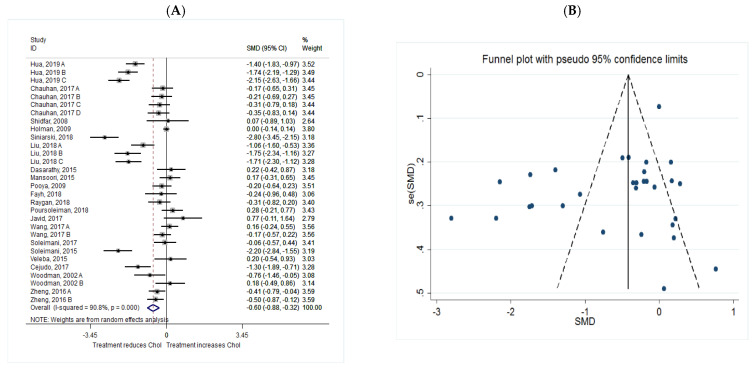
(**A**) The effect of *n*-3 on Chol; (**B**) funnel plot examining the publication bias of Chol and *n*-3 supplementation.

The common SMD from 20 studies was −0.60 (95% CI: −0.88 to −0.32, *p* = 0.00) based on a random effect model, with significant heterogeneity between studies (I^2^ = 90.8%, *p* = 0.00) (Figure 6A). Further investigation detected 7 outliers with large effect sizes (Hua, 2019 B, SMD = −2.57; Hua, 2019 C, SMD = −3.50; Siniarski, 2018, SMD = −4.97; Liu, 2018 B, SMD = −2.60; Liu, 2018 C, SMD = −2.51; Poursoleiman, 2018, SMD = 3.09; Soleimani, 2017, SMD = −3.61), therefore they were removed from the analysis. Removing these studies resulted in a reduction in effect size (SMD = −0.35, 95% CI: −0.58 to −0.13), but heterogeneity still remained significant although a reduction in heterogeneity was observed (I^2^ = 81.3%, *p* = 0.00). According to the shape of the funnel plot, we considered that there was no obvious publication bias among the included studies with cholesterol as an outcome measure (Figure 6B).

##### LDL

**Figure 7:**
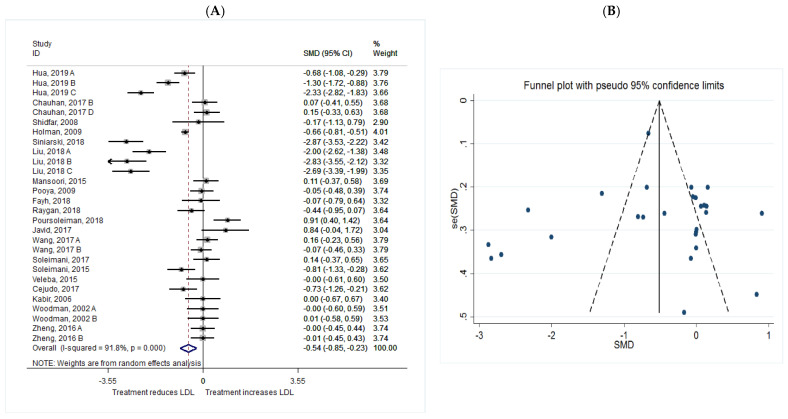
(**A**) The effect of *n*-3 on LDL; (**B**) funnel plot examining the publication bias of LDL and *n*-3 supplementation.

The common SMD from 20 studies was −0.54 (95% CI: −0.85 to −0.23, *p* = 0.001) based on a random effect model, with significant heterogeneity between studies (I^2^ = 91.8%, *p* = 0.00) (Figure 7A). Further investigation detected 7 outliers with large effect sizes (Hua, 2019 C, SMD = −3.65; Siniarski, 2018, SMD = −4.75; Liu, 2018 A, SMD = −2.98; Liu, 2018 B, SMD = −4.67; Liu, 2018 C, SMD = −4.39; Poursoleiman, 2018, SMD = 2.96; Javid, 2017, SMD = 2.82). Therefore, these studies were removed from further analyses in this outcome. The removal of these studies resulted in a reduction in effect size (SMD = −0.22, 95% CI: −0.42 to −0.03), but heterogeneity remained significant although a reduction in heterogeneity was observed (I^2^ = 74.7%, *p* = 0.00). 

Publication bias regarding studies of LDL was observed using visual inspection of funnel plot, however, the results of a Begg’s and Egger’s asymmetry test were not significant (*p* > 0.05). Trim and fill analysis was therefore conducted to correct the bias imputing three hypothetical studies. The results were significant even after trim and fill analysis (SMD = −0.662; 95% CI: −0.96 to −0.35; *p* = 0.00) (Figure 7B).

##### HDL

**Figure 8:**
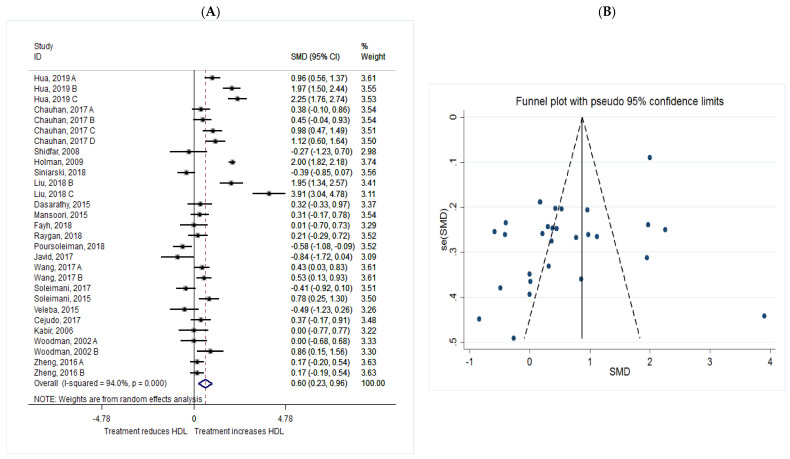
(**A**): The effect of *n*-3 on HDL; (**B**) funnel plot examining the publication bias of HDL and *n*-3 supplementation.

The common SMD from 20 studies was 0.60 (95% CI: 0.23 to 0.96, *p* = 0.001) based on a random effect model, with significant heterogeneity between studies (I^2^ = 94.0%, *p* = 0.00) (Figure 8A). Further investigation detected 7 outliers with large effect sizes (Hua, 2019 B, SMD = 2.44; Hua, 2019 C, SMD = 2.92; Holman, 2009, SMD = 2.50; Liu, 2018 B, SMD = 2.41; Liu, 2018 C, SMD = 5.76; Poursoleiman, 2018, SMD = −2.36; Javid, 2017, SMD = −2.50). These studies were therefore removed from further analyses of the HDL as an outcome. Removal of these studies resulted in a reduction in effect size (SMD = 0.32, 95% CI: 0.14 to 0.51), but heterogeneity still remained significant although considerable reduction in heterogeneity was observed (I^2^ = 64.0%, *p* = 0.00). According to the shape of funnel plot, we considered that there was no obvious publication bias among the included studies (Figure 8B).

Sub-group analysis of different dosage of *n*-3 consumption (<1000, 1000–200, or >2000 mg/d) showed that *n*-3 consumption in all the 3 dose sub-groups could significantly reduce TG and total-cholesterol levels. LDL and HDL levels were also significantly changed by *n*-3 consumption of more than 1000 mg/d. Moreover, sub-group analysis of duration of *n*-3 consumption (≤8 week/>8 week) showed that *n*-3 consumption for both ≤8 and >8 weeks could significantly reduce TG and total cholesterol levels and increase HDL level; however, the significant reduction in LDL level occurred only when *n*-3 was consumed for more than 8 weeks (Table 3).

#### 2.2.3. Inflammatory Markers

##### TNF-α

**Figure 9:**
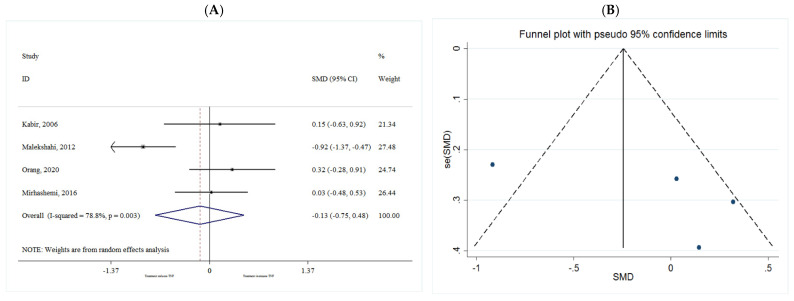
(**A**) The effect of *n*-3 on TNF-α; (**B**) funnel plot examining the publication bias of TNF-α and *n*-3 supplementation.

The common SMD from 4 studies was −0.13 (95% CI: −0.75 to 0.48, *p* = 0.668) based on a random effect model, with significant heterogeneity between studies (I^2^ = 78.8%, *p* = 0.003) (Figure 9A). Further investigation detected no outliers. 

Publication bias regarding studies of TNF-α was observed using visual inspection of funnel plot, however, the results for Begg’s and Egger’s asymmetry test were not significant (*p* > 0.05). Therefore, trim and fill analysis were conducted to correct the bias imputing two hypothetical studies. The results were non-significant even after trim and fill analysis (SMD = −0.482; 95% CI: −1.05 to 0.08; *p* = 0.096) (Figure 9B).

##### CRP

**Figure 10:**
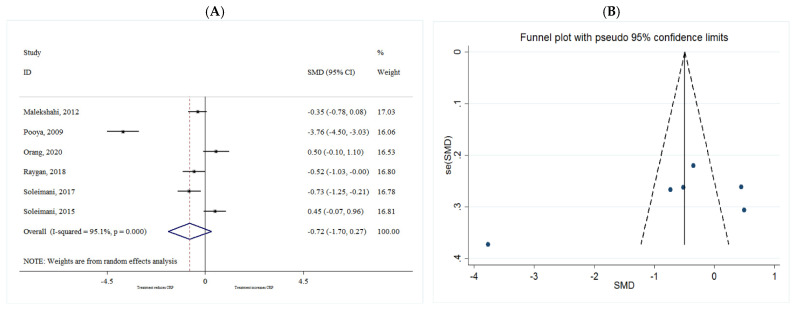
(**A**) The effect of *n*-3 on CRP; (**B**) funnel plot examining the publication bias of CRP and *n*-3 supplementation.

The common SMD from 6 studies was −0.72 (95% CI: −1.70 to 0.27, *p* = 0.156) based on a random effect model, with significant heterogeneity between studies (I^2^ = 95.1%, *p* = 0.00) (Figure 10A). Further investigation detected no outliers. Furthermore, according to the shape of the funnel plot, it was considered that there was no obvious publication bias among the included studies (Figure 10B).

#### 2.2.4. Anthropometric Parameters

##### Weight

**Figure 11:**
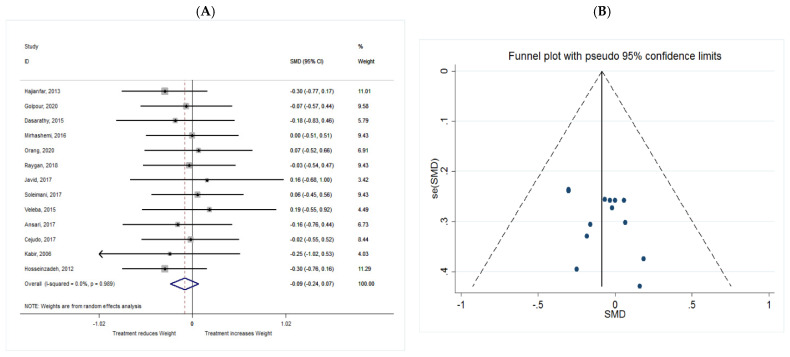
(**A**) The effect of *n*-3 on weight; (**B**) funnel plot examining the publication bias of weight and *n*-3 supplementation.

The common SMD from 13 studies was −0.09 (95% CI: −0.24 to 0.07, *p* = 0.280) based on a random effect model, with no heterogeneity between studies (I^2^ = 0.0%, *p* = 0.989) (Figure 11A). 

Publication bias regarding studies of weight was observed using visual inspection of funnel plot, however, the results for Begg’s and Egger’s asymmetry test were not significant (*p* > 0.05). Trim and fill analysis was conducted to correct the bias imputing two hypothetical studies. The results were not significant even after trim and fill analysis (SMD = −0.108; 95% CI: −0.25 to 0.042; *p* = 0.157) (Figure 11B).

##### BMI

**Figure 12:**
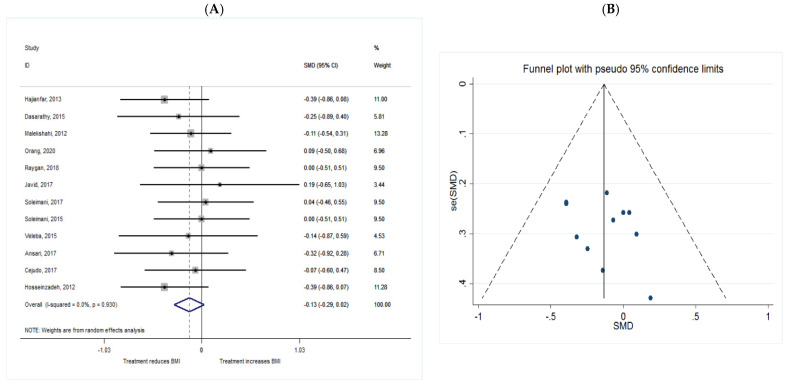
(**A**) The effect of *n*-3 on BMI; (**B**) funnel plot examining the publication bias of BMI and *n*-3 supplementation.

The common SMD from 12 studies was −0.13 (95% CI: −0.29 to 0.02, *p* = 0.093) based on a random effect model, with no heterogeneity between studies (I^2^ = 0.0%, *p* = 0.930) (Figure 12A). According to the shape of funnel plot, we considered that there was no obvious publication bias among the included studies (Figure 12B).

## 3. Discussion

The beneficial effects of *n*-3 supplementation on metabolic biomarkers have been previously investigated in several studies, including our own, in T2DM [25,43,44]. The present review has systematically analyzed RCTs to further clarify the effects of *n*-3 consumption on glycemic factors, body weight, lipid profile, and inflammatory biomarkers in patients with T2DM. The results show that consuming *n*-3 significantly improve glycemic factors (FBS, HbA1c, and HOMA.IR) and lipid profile (TG, total cholesterol, LDL, and HDL) in patients with T2DM. However, in the small number of sample studies investigated reductions in weight, BMI, and inflammatory biomarkers (TNF-α and CRP) were not statistically significant. 

It has long been known that membrane phospholipid PUFAs composition is associated with insulin sensitivity [45]. The beneficial effects of *n*-3 fatty acids on glycemic control and glucose homeostasis are likely to involve several mechanisms. With increased incorporation of *n*-3 fatty acids into cellular membranes, via supplementation, membrane fluidity and several cell membrane and intracellular receptors, which regulate cellular signaling and gene expression, can be affected leading to increased insulin sensitivity [46]. For example, EPA and DHA can increase GLUT1 and GLUT4 translocation respectively and the transport of glucose [47,48]. Furthermore, *n*-3 fatty acids may improve glucose homeostasis through regulating inflammation [48,49]. In addition, studies in animal models have shown that *n*-3 fatty acids improve insulin sensitivity and glucose homeostasis by influencing the insulin signaling pathway [50,51,52]. The results of the present meta-analysis show that *n*-3 supplementation can significantly improve the glycemic response in patients with T2DM. The results are consistent with those of a previous meta-analysis conducted by O’Mahoney et al. [43]. They showed that *n*-3 PUFAs supplementation can produce favorable improvement in glycaemia. Importantly, in the present investigation, in a sub-group analysis, we found that consuming a dose of 1000–2000 mg/d *n*-3 supplement for more than 8 weeks can improve the FBS level and HOMA.IR index. A previous study demonstrated that *n*-3 fatty acids supplementation at high doses (12 g of fish oil for 6 weeks) increased glycerol gluconeogenesis by 32%, which could contribute to a deterioration of glycemic control during long-term treatment [53]. Therefore, we suggest based on our analysis the optimal dosage of *n*-3 supplementation, for improving glycemic and related parameters is 1000–2000 mg/d.

It is well established that T2DM is associated with dyslipidemia (4) and that *n*-3 intake has long been indicated in the treatment of hyperlipidemia [30,31]. As cell membrane fatty acids play an important role in signal transduction, and *n*-3 fatty acids are capable of modifying gene expression, it is thought that the dramatic lipid-altering effects of *n*-3 fatty acids are mediated via this mechanism [54]. More specifically, *n*-3 fatty acids can modulate the function of peroxisome proliferator–activated receptors (PPARs) and sterol regulatory element-binding proteins (SREBPs), both of which are involved in lipid homeostasis [55] and have been reviewed in detail elsewhere [54]. We observed a significant reduction in triglycerides, total cholesterol, and LDL levels and significant increase in HDL level in response to *n*-3 supplementation in T2DM. These findings are similar to the results of previous meta-analyses [56,57,58,59]. Our sub-group analysis however also revealed that *n*-3 supplementation of greater than 1000 mg/d for more than 8 weeks can significantly improve the lipid profile in patients with T2DM.

In relation to inflammation TNF-α and IL-6 can impair insulin signaling and action by post-translational modulation of insulin receptor substrates [60]. Furthermore, TNF-α can induce lipolysis in fat cells leading to increased free fatty acids [61] and the adipocytokines have inflammatory effects, e.g., pro-inflammatory resistin and leptin [25,62]. Clinical trials indicate that *n*-3 fatty acid supplementation can affect the serum concentrations of inflammatory biomarkers, including TNF-α and serum CRP levels. A direct mechanism through which *n*-3 PUFA can decrease inflammation includes rapid effects on the regulation of transcription factors [63,64], and indirect modes of actions including the production of, e.g., five-series eicosanoids [65] and inflammation-resolving lipid mediators and suppression of acute phase reactants [66,67]. In a study conducted by Rangel-Huerta et al., they reviewed 26 RCTs which supplemented *n*-3 FAs over 10 years; ten of the reported trials were undertaken in healthy and the remainder in chronic diseases participants [68]. In the studies of healthy participants, they found that *n*-3 supplementation generally had no effect on inflammatory biomarkers, which may have been due to low circulating levels. Among the cardiovascular disease patients, the level of CRP and IL-6 was generally decreased after 12 weeks of the *n*-3 supplement. In contrast in the large meta-analysis by Li et al. [69] which included 68 RCTs (total of 4601 subjects) they found that marine-derived *n*-3 PUFA (EPA, DHA, fish oil) significantly decreased CRP, TNF-α and IL-6 in healthy (particularly older subjects) and in patients with chronic non-autoimmune disease. They also reported that the effects were associated with treatment dose and duration. In the current analysis however, we found that *n*-3 supplementation did not significantly decrease inflammatory biomarkers. This may in-part be due to low number of included studies, the variable levels of inflammatory biomarkers reported, use of drug treatments, background diet and relatively low dose used in some of the studies. This meta-analysis and other studies discussed consistently show that *n*-3 supplementation effects on metabolic or inflammatory markers depends on dose and time. Most likely this is a function of the time required to fully incorporate fatty acids into membrane phospholipids and affect dysregulated homeostatic mechanisms. 

There is evidence that increased intakes of *n*-3 fatty acids can reduce body fat in humans, but human studies are relatively few and have generally been conducted over short time periods with small sample sizes [70]. The mechanisms by which *n*-3 PUFA reduce body fat are not well understood. There is evidence from both human [71] and animal [72,73] studies that suggest that these fatty acids may contribute to improvements in body composition by suppressing appetite and promoting apoptosis of adipocytes [73]. Moreover, there is considerable evidence from animal studies indicating that the effects of *n*-3 PUFA on body weight and body fat are mediated by altering the expression of genes involved in the regulation of fat metabolism in several tissues. However, the effect of *n*-3 supplementation on body weight and BMI was not significant in the present analysis. More studies to increase sample size, better controlled inclusion and exclusion criteria for weight or longer duration and higher dosage of *n*-3 supplementation might uncover significant effects of *n*-3 supplementation on weight in T2DM. 

There have been several other meta-analysis studies of the effects of *n*-3 in T2DM. Chen et al. (2015) [74] reported no significant difference in glucose control between placebo and *n*-3 supplementation but did report beneficial effects on lipid profile in their meta-analysis. In the meta-analysis by Brown et al. (2019) [75] they found that neither *n*-3, *n*-6 or total PUFA supplementation affects the development or treatment of diabetes. Importantly Brown et al. investigated both diet intake and supplementation studies. It is well known that dietary intake analysis is not necessarily precise (in contrast our data were obtained directly from supplementation RCTs only) and they also state that their data included a high number of RCTs at risk of bias. In the meta-analysis undertaken by Natto et al. (2019) [76] they suggested that *n*-3 supplementation may improve metabolic or inflammatory markers, e.g., TNF-α in patients with diabetes or cardiovascular disease, but their data was not conclusive, and furthermore they did not explore the effects of dose or time. Gao et al. (2020) [77] in their meta-analysis found no effect of fish oil supplementation on glucose metabolism parameters but did observe a positive effect on lipid profile markers. The difference in glycemic parameters we observed in the present study may be linked to the different supplement sources included in our meta-analysis. Furthermore, in some of the RCTs included in previous studies there may have been dietary components that track with *n*-3 intake which could have disrupted any beneficial effects. We included a greater number of *n*-3 supplementation RCTs in our meta-analysis compared with most previous meta-analysis studies, which may also account for the glycemic parameter differences between some previous studies and present analysis.

The present study has some limitations. Firstly, the number of studies evaluating the effect of *n*-3 supplementation on inflammatory markers and body weight was low. Secondly, different *n*-3 fatty acids (parent ALA and it’s metabolic products EPA and DHA) supplementation has been used in different studies i.e., marine-derived (EPA, DHA and fish oil) and plant-derived (ALA, flaxseed). Although it is generally accepted that ALA exerts its effects via conversion to EPA [78], it is also possible ALA has biological effects in T2DM without conversation to EPA [79]. Thirdly, significant heterogeneity was found in most of the analyzed parameters, and the source of the heterogeneity was not explored further. We also only used random-effects models to address heterogeneity, which may have affected the strength and extrapolation of our conclusions. In addition, it is becoming clear that the effects of *n*-3 fatty acids supplementation in T2DM populations differ depending on their ethnic and dietary backgrounds [44]. Finally, the effects of a more balanced intake of *n*-3 and *n*-6 PUFAs on metabolic and inflammatory biomarkers [45,79,80,81,82] with or without probiotics [83] on T2DM are also now warranted.

## 4. Methods

This study followed the Preferred Reporting Items for Systematic Reviews and Meta-Analyses (PRISMA) guidelines. The study protocol was registered prospectively in PROSPERO (CRD42021250440).

### 4.1. Search Strategy

Online databases PubMed, Embase, Web of Science, and Science Direct were searched until 2021 for studies that investigated the effects of omega-3 supplementation on metabolic biomarkers and body weight in patients with T2DM. The following search terms were used: (Omega-3 OR *n*-3 OR fish oil) AND (diabetes OR type 2 diabetes OR T2D OR T2DM) AND (FBS OR fasting blood glucose OR glycemic OR glucose OR Insulin OR HOMA.IR OR A1c OR HbA1c OR lipid profile OR TG OR Cholesterol OR LDL OR HDL OR triglyceride OR CRP OR TNF-α OR weight OR BMI OR Body Mass Index). Our search was restricted to studies published in the English language.

### 4.2. Study Selection and Inclusion and Exclusion Criteria

The following criteria were used to identify eligible studies: (i) randomized placebo-controlled trials with either parallel or cross-over design, (ii) investigation of the effects of *n*-3 on glycemic factors in patients with T2DM, (iii) investigation of the effects of *n*-3 on lipid profile in patients with T2DM, (iv) investigation of the effects of *n*-3 on inflammatory bio-markers in patients with T2DM, (v) investigation of the effects of *n*-3 on BMI or body weight in patients with T2DM and (vi) providing sufficient information on the baseline and end-trial metabolic bio-markers, weight, and/or BMI in both *n*-3 and control groups. Exclusion criteria were (i) animal studies, (ii) observational studies, (iii) uncontrolled studies, and (iv) lack of sufficient/relevant information on the baseline or end-trial blood biomarkers. 

### 4.3. Data Extraction

The following data were abstracted: (1) first author’s name; (2) year of publication; (3) number of participants in the omega-3 and control groups; (4) dose of omega-3 supplement; and (5) treatment duration. 

### 4.4. Quality Assessment

A systematic assessment of bias in the included studies was performed using the Cochrane criteria. The items used for the assessment of each study were as follows: adequacy of sequence generation, allocation concealment, blinding, addressing of dropouts (incomplete outcome data), selective outcome reporting, and other potential sources of bias. According to the recommendations of the Cochrane Handbook, a judgment of “yes” indicated a low risk of bias, while “no” indicated high risk of bias. Labeling an item as “unclear” indicated an unclear or unknown risk of bias.

### 4.5. Statistical Analysis

The mean changes (mean values and SD) in fasting blood sugar (FBS), glycated hemoglobulin (HbA1c), homeostatic model assessment of insulin resistance (HOMA.IR), insulin, malonaldehyde (MDA), c-reactive protein (CRP), tumor necrosis factor-alpha (TNF-α), triglycerides (TG), cholesterol, low density lipoprotein (LDL), high density lipoprotein (HDL), weight, and body mass index (BMI) for each study were calculated. Statistical analysis was conducted using Stata 16.0 (Stata Corp, College Station, Texas, USA). The heterogeneity among studies was evaluated by Cochran heterogeneity test and I^2^ statistic. *p*-values of <0.05 or I^2^ of >50% indicated that heterogeneity existed among studies. Otherwise, homogeneity of those studies was indicated. The standardized mean difference (SMD) of each study along with its 95% CI was calculated. Sensitivity analysis was carried out to check the influence of one single study removal on overall effect size. Additionally, funnel plots were conducted for assessing the publication bias of included literatures and we could assess the publication bias by seeing whether their shapes were of any obvious asymmetry. To find the studies with outlier SMD, a series of sensitivity analyses was conducted and in the case of being outlier a second meta-analysis was performed after removing the outlier study.

## 5. Conclusions

The present meta-analysis and review found that *n*-3 supplementation can regulate the glycemic response and lipid profile in patients with T2DM. Furthermore *n*-3 supplementation may provide beneficial effects on inflammatory biomarkers and body weight if used at a specific dose and duration. The preferred dose and duration for patients with T2DM is 1000–2000 mg/d for more than 8 weeks. More studies are needed to fully evaluate the effect of the different types of *n*-3 fatty acids i.e., fish-oil, and EPA and DHA and plant-derived ALA supplements on T2DM metabolic and inflammatory biomarkers. The effectiveness of different doses and durations of such interventions in T2DM should be compared and should include T2DM populations with different dietary and ethnic backgrounds.

## Figures and Tables

**Table 1:** Characteristics of included studies.

N	ID		Supplement Type	Duration (Weeks)	Dose (mg/d)	Analysis										
						Glycemic Factors			Lipid Profile				Inflammatory Markers		Weight	BMI
						FBS	HbA1c	HOMA.IR	TG	Chol	LDL	HDL	TNF-α	CRP		
1	Hajianfar, [14]		*n*-3 capsules	8	-										✓	✓
2	Golpour, [15]		*n*-3 capsules	10	2700	✓	✓	✓							✓	✓
3	Dasarathy, [16]		EPA/DHA supplement	48	3600	✓	✓	✓		✓		✓			✓	✓
4	Mirhashemi, [17]		flaxseed oil	12	1000								✓		✓	
5	Orang, [18]		*n*-3 capsules	12	2000	✓	✓	✓					✓	✓	✓	✓
6	Raygan, [19]		fish oil	12	1000	✓		✓	✓	✓	✓	✓		✓	✓	✓
7	Javid, [20]		*n*-3 capsules	8	2000	✓	✓		✓	✓	✓	✓			✓	✓
8	Soleimani, [21]		flaxseed oil	12	1000	✓	✓	✓	✓	✓	✓	✓		✓	✓	✓
9	Soleimani, [22]		flaxseed oil	12	1000	✓			✓	✓	✓	✓		✓		
10	Veleba, [23]		EPA + DHA concentrate	24	5000	✓	✓		✓	✓	✓	✓			✓	✓
11	Ansari, [24]		*n*-3 capsules	10	3750	✓	✓	✓							✓	✓
12	Jacobo-Cejudo, [25]		fish-oil	24	520	✓	✓	✓	✓	✓	✓	✓			✓	✓
13	Kabir, [26]		fish-oil	8	3000	✓	✓		✓		✓	✓	✓		✓	
14	Hosseinzadeh, [27]		*n*-3 capsules	8	2000										✓	✓
15	Malekshahi, [28]		*n*-3 capsules	8	2714								✓	✓		✓
16	Hua, [29]	A	Fish oil	4	2000	✓	✓	✓	✓	✓	✓	✓				
		B		8		✓	✓	✓	✓	✓	✓	✓				
		C		12		✓	✓	✓	✓	✓	✓	✓				
17	Chauhan, [30]	A	*n*-3 capsule	6	1000	✓			✓	✓		✓				
		B		12	2000	✓	✓		✓	✓	✓	✓				
		C		6	1000	✓			✓	✓		✓				
		D		12	2000	✓	✓		✓	✓	✓	✓				
18	Shidfar, [31]		Purified *n*-3	10	2000	✓	✓		✓	✓	✓	✓				
19	Udupa, [32]		*n*-3 soft gels	13	300	✓	✓									
20	Liu, [33]	A	fish oil	4	3650	✓	✓	✓	✓	✓	✓					
		B		8		✓	✓	✓	✓	✓	✓	✓				
		C		12		✓	✓	✓	✓	✓	✓	✓				
21	Mansoori, [34]		fish oil	8	1850	✓		✓	✓	✓	✓	✓				
22	Pooya, [35]		*n*-3 capsules	8	2714	✓	✓			✓	✓			✓		
23	Fayh, [36]		*n*-3 capsules	8	300	✓	✓		✓	✓	✓	✓				
24	Poursoleiman, [37]		*n*-3 soft gels	6	2000	✓			✓	✓	✓	✓				
25	Wang, [38]	A	fish oil	12	4000	✓	✓	✓	✓	✓	✓	✓				
		B		24		✓	✓	✓	✓	✓	✓	✓				
26	Sarbolouki, [39]		purified EPA	12	2000	✓	✓	✓								
27	Woodman, [40]	A	purified EPA	6	4000	✓	✓		✓	✓	✓	✓				
		B	purified DHA	6	4000	✓	✓		✓	✓	✓	✓				
28	Zheng, [22]	A	fish oil	12	2000	✓	✓	✓		✓	✓	✓				
		B		26		✓	✓	✓		✓	✓	✓				
29	Holman, [41]		*n*-3 capsules	28	2000		✓			✓	✓	✓				
30	Siniarski, [42]		juice box	12	2000				✓	✓	✓	✓				

**Table 2:** Pooled estimates of *n*-3 effects on glycemic factors within dosage and duration subgroups.

Variable	Group	No. of Comparisons	SMD (95% CI)	*p*-Value	I^2^ (%)	*p*-Heterogeneity
FBS	Total	34	−0.36 (−0.71, −0.01)	0.047	92.9	0.000
	Intervention dosage (mg/d)					
	≤1000	8	−0.20 (−0.83, 0.42)	0.084	40.9	0.084
	1000–2000	13	−0.33 (−0.57, −0.09)	0.008 *	69.0	0.000
	≥2000	13	−1.40 (−1.42, 0.63)	0.448	96.9	0.000
	Intervention duration (w)					
	≤8	14	−0.21 (−0.96, 0.54)	0.588	95.6	0.000
	>8	20	−0.40 (−0.75, −0.05)	0.026 *	92.9	0.000
HbA1c	Total	29	−0.74 (−1.13, −0.35)	0.047	94.8	0.000
	Intervention dosage (mg/d)					
	≤1000	5	−0.66 (−1.11, −0.20)	0.005 *	68.4	0.013
	1000–2000	11	−0.75 (−1.37, −0.14)	0.016 *	95.9	0.000
	≥2000	13	−0.81 (−1.58, −0.03)	0.042 *	95.4	0.000
	Intervention duration (w)					
	≤8	10	−0.69 (−1.29, −0.08)	0.025 *	90.4	0.000
	>8	19	−0.77 (−1.25, −0.29)	0.002 *	95.3	0.000
HOMA.IR	Total	19	−0.58 (−1.13, −0.35)	0.038	95.6	0.000
	Intervention dosage (mg/d)					
	≤1000	3	0.32 (−0.96, 0.32)	0.324	77.5	0.012
	1000–2000	8	−0.93 (−1.71, −0.16)	0.019 *	95.6	0.000
	≥2000	8	−0.33 (−1.40, 0.74)	0.546	96.6	0.000
	Intervention duration (w)					
	≤8	5	0.16 (−1.28, 1.60)	0.828	97.6	0.000
	>8	14	−0.84 (−1.42, −0.26)	0.005 *	94.5	0.000

Note: * Shows statistical significance level.

**Table 3:** Pooled estimates of *n*-3 effects on lipid profile within dosage and duration subgroups.

Variable	Group	No. of Comparisons	SMD (95% CI)	*p*-Value	I^2^ (%)	*p*-Heterogeneity
TG	Total	26	−0.27 (−0.37, −0.18)	0.038	0.0	0.958
	Intervention dosage (mg/d)					
	≤1000	7	−0.23 (−0.43, 0.03)	0.023 *	0.0	0.851
	1000–2000	10	−0.32 (−0.47, −0.17)	0.000 *	0.0	0.621
	≥2000	9	−0.24 (−0.42, −0.07)	0.006 *	0.0	0.958
	Intervention duration (w)					
	≤8	13	−0.23 (−0.37, −0.08)	0.002 *	0.0	0.909
	>8	13	−0.23 (−0.45, −0.18)	0.000 *	0.0	0.904
CHOL	Total	30	−0.60 (−0.88, −0.32)	0.038	90.8	0.000
	Intervention dosage (mg/d)					
	≤1000	7	−0.62 (−1.16, −0.08)	0.024 *	85.3	0.000
	1000–2000	13	−0.66 (−1.14, −0.18)	0.007 *	94.2	0.000
	≥2000	10	−0.49 (−0.95, −0.03)	0.037 *	86.0	0.000
	Intervention duration (w)					
	≤8	13	−0.50 (−0.93, −0.07)	0.024 *	88.1	0.000
	>8	17	−0.67 (−1.05, −0.29)	0.001 *	92.3	0.000
LDL	Total	28	−0.54 (−0.85, −0.23)	0.047	91.8	0.000
	Intervention dosage (mg/d)					
	≤1000	6	−0.31 (−0.65, 0.03)	0.073	58.2	0.035
	1000–2000	12	−0.51 (−1.01, −0.02)	0.042 *	93.9	0.000
	≥2000	10	−0.72 (−1.38, −0.07)	0.029 *	93.1	0.000
	Intervention duration (w)					
	≤8	12	−0.43 (−0.98, 0.13)	0.132	91.8	0.000
	>8	16	−0.62 (−1.01, −0.23)	0.002 *	92.2	0.000
HDL	Total	29	0.60 (0.23, 0.96)	0.001	94.0	0.000
	Intervention dosage (mg/d)					
	≤1000	7	0.27 (−0.01, 0.55)	0.063	48.8	0.069
	1000–2000	13	0.63 (0.04, 1.22)	0.036 *	96.0	0.000
	≥2000	9	0.81 (0.14, 1.49)	0.001 *	91.1	0.000
	Intervention duration (w)					
	≤8	12	0.52 (0.03, 1.01)	0.036 *	88.9	0.000
	>8	17	0.65 (0.15, 1.16)	0.012 *	95.5	0.000

Note: * Shows statistical significance level.

## Data Availability

Published RCTs and PROSPERO (CRD42021250440).
